# Repeated Vowel Production Affects Features of Neural Activity in Sensorimotor Cortex

**DOI:** 10.1007/s10548-018-0673-4

**Published:** 2018-09-20

**Authors:** E. Salari, Z. V. Freudenburg, M. J. Vansteensel, N. F. Ramsey

**Affiliations:** 1 Brain Center Rudolf Magnus, Department of Neurology and Neurosurgery, University Medical Center Utrecht, Utrecht, The Netherlands; 2 University Medical Center Utrecht, Room G03 1.24, Heidelberglaan 100, P.O. Box 85500, 3508 GA Utrecht, The Netherlands

**Keywords:** Sensorimotor cortex, Movement, Repetition, ECoG, Speech, Brain–computer interface

## Abstract

The sensorimotor cortex is responsible for the generation of movements and interest in the ability to use this area for decoding speech by brain–computer interfaces has increased recently. Speech decoding is challenging however, since the relationship between neural activity and motor actions is not completely understood. Non-linearity between neural activity and movement has been found for instance for simple finger movements. Despite equal motor output, neural activity amplitudes are affected by preceding movements and the time between movements. It is unknown if neural activity is also affected by preceding motor actions during speech. We addressed this issue, using electrocorticographic high frequency band (HFB; 75–135 Hz) power changes in the sensorimotor cortex during discrete vowel generation. Three subjects with temporarily implanted electrode grids produced the /i/ vowel at repetition rates of 1, 1.33 and 1.66 Hz. For every repetition, the HFB power amplitude was determined. During the first utterance, most electrodes showed a large HFB power peak, which decreased for subsequent utterances. This result could not be explained by differences in performance. With increasing duration between utterances, more electrodes showed an equal response to all repetitions, suggesting that the duration between vowel productions influences the effect of previous productions on sensorimotor cortex activity. Our findings correspond with previous studies for finger movements and bear relevance for the development of brain-computer interfaces that employ speech decoding based on brain signals, in that past utterances will need to be taken into account for these systems to work accurately.

**Electronic supplementary material:**

The online version of this article (10.1007/s10548-018-0673-4) contains supplementary material, which is available to authorized users.

## Introduction

The execution of everyday voluntary body movements generally occurs without effort and is the result of the concerted action of different neural processes and brain areas. The sensorimotor cortex is known to play a central role in the different aspects of the generation of movement, such as the control of body part positions, the velocity and direction of movements, applied force and the planning of motor actions (Tanji and Evarts 1976; Georgopoulos et al. 1982, 1992; Donoghue et al. 1998; Moran and Schwartz 1999; Wang et al. 2007; Truccolo et al. 2008). However, for subjects suffering from severe forms of paralysis, even the most common forms of movements, such as those involved in speech and communication can sometimes be completely absent (American Congress of Rehabilitation Medicine 1995; Smith and Delargy 2005; Posner et al. 2007). To restore communication in these subjects, brain-computer-interface (BCI) systems are being developed (Wolpaw et al. 2002). These systems may convert neural activity into written or spoken computer output, and sensorimotor cortex activity related to speech has been shown useful in an attempt to identify, from the neural signals, which sound or word a user may want to communicate (Kellis et al. 2010; Mugler et al. 2014; Herff et al. 2015; Ramsey et al. 2017). These attempts usually rely on the assumption that each specific sound or word is associated with a unique neural signature. Imaging and patient studies, however, have shown that repeating a movement in a discrete way (with short pauses between each movement) may involve different brain areas than performing the same movements in a continuous way (without short pauses between each movement; Kennerley et al. 2002; Spencer et al. 2003; Miall and Ivry 2004; Schaal et al. 2004), even though the movements are almost identical. Moreover, there is evidence for a non-linear relationship between movement-performance and neural activity in the sensorimotor cortex. Various studies have suggested that previous actions influence the neural activity associated with subsequent actions, if spaced close enough together (Miezin et al. 2000; Soltysik et al. 2004). Indeed, during repeated finger movements, the amplitude of sensorimotor neural activity, as measured with fMRI and electrocorticography (ECoG), was shown to decline over repetitions, despite equal movement output (Hermes et al. 2012b; Siero et al. 2013; for a comparison between BOLD and ECoG see: Logothetis et al. 2001; Hermes et al. 2012a; Siero et al. 2014).

Importantly, the studies mentioned above focused on hand and finger movements and it remains to be determined whether the observed complex and non-linear relationship between movement and underlying neural activity is a general feature of the sensorimotor cortex, or whether it is specific to the areas involved in hand movement. Especially relevant in this respect is our previous finding that different parts of the sensorimotor cortex show different response profiles to the same *speech* movement. Some cortical foci show sustained neural activity during a sustained motor speech action whereas in other locations responses are transient during the same movement (Salari et al. 2018). This finding indicates that the relationship between neural activity and overt speech behaviour differs between subareas of the sensorimotor cortex. It could be speculated that the presence, or absence, of a non-linear relationship between neural responses and behavioural output during repeated movements is specific for cortical foci as well.

With the current study, we aimed to obtain a better understanding of the link between speech pronunciation and underlying sensorimotor cortex activity. This is of interest for BCIs that employ neural signal changes related to (attempted) speech. If the neural signal associated with a specific (attempted) pronunciation would be affected by previous speech actions, the same word or sound may be related to a diversity of neural signatures, which have to be taken into account for a sensorimotor-speech-BCIs to function accurately.

In this study, we investigated the relationship between repeated orofacial movements during speech, and sensorimotor brain activity. We recorded neural signals in three subjects while they pronounced the same vowel multiple times, at different repetition rates. Neural activity was recorded with subdural ECoG electrodes, which allows for recording at high temporal resolution and with high spatial specificity (Siero et al. 2014). We focused on frequencies in the range of 75–135 Hz, which are known to have a spatially specific relationship with (speech and articulator) movements (Crone et al. 1998; Miller et al. 2007; Bouchard et al. 2013), and which are thought to reflect underlying neural population firing (Manning et al. 2009; Miller et al. 2009; Ray and Maunsell 2011). We focused mostly on the ventral parts of the sensorimotor cortex as this area has previously been shown to be responsible for the generation of speech movements (Penfield and Boldrey 1937; Crone et al. 2001; Towle et al. 2008; Pei et al. 2011; Bouchard et al. 2013) and has been the focus of BCI-studies for the classification of speech sounds (see for instance Kellis et al. 2010; Mugler et al. 2014; Herff et al. 2015; Ramsey et al. 2017) and articulator movements (Bleichner et al. 2015).

## Method

### Subjects

Subjects included in this study (n = 3, 2 females, 19, 41 and 30 years old respectively) were implanted with subdural clinical ECoG electrodes for epilepsy treatment at the University Medical Center Utrecht. All subjects had an additional high-density (HD) electrode grid placed over the sensorimotor cortex (SMC; left for subject A & B and right for subject C). These grids were exclusively placed for research purposes with the subject’s consent, over an area that was not clinically relevant. For subject A & B, the inter-electrode distance of the HD grid was 4 mm with an exposed electrode diameter of 1 mm for subject A and 1.17 mm for subject B. For subject C, the inter-electrode distance was 3 mm with an exposed electrode diameter of 1 mm. Only the HD electrodes were used for the current analysis.

This research was approved by the ethics committee of the University Medical Center Utrecht. All participants gave written informed consent in accordance with the Declaration of Helsinki (2013).

### Task

Participants were asked to produce the /i/ vowel repeatedly at different rates (see below), guided by instructions that were visually presented on a computer screen that was placed at a distance of approximately 1 m from the participant. A trial started with an indication of the production speed by a visual cue. Subsequently, to guide the participants in producing the sound at the correct speed, the letters ‘ie’, corresponding in Dutch to the /i/ sound, were repeatedly visually presented for 300 ms at a rate of 5, 4, or 3 times in 3 s (1.66, 1.33 and 1 Hz). These repetition rates were chosen as they were relatively easy to perform (not too slow or too fast) and because previous research for finger movements has shown that repetition effects are mostly apparent at rates of 1 Hz or higher (Hermes et al. 2012b). During the inter-trial interval (1800 ms), a fixation cross was presented. Trials of different rates were randomized and each rate was repeated 26 times, divided over two recording sessions. Any trial for which the number of pronunciations was incorrect, was excluded from the analyses.

### Data Acquisition & Preprocessing

Brain data was recorded and preprocessed as described previously (Salari et al. 2018). In short, ECoG data was recorded (number of electrodes: 64 for subject A and 128 for subject B & C) at a sampling frequency of 512 Hz, 2048 Hz (subject A; Micromed, Treviso, Italy), or 2000 Hz (subject B & C; Blackrock Microsystems LLC, Salt Lake City, USA). Different sampling frequencies were used due to the availability of different clinical and research recording setups and the possibility, or not, to choose the most optimal sampling frequency for the current study. For subject A, the data obtained at the highest sampling frequency was down sampled such that the sampling frequencies of all datasets of subject A were the same. Electrodes in the region of interest (sensorimotor cortex) were identified by visual inspection of the electrode positions (as determined by using a post-implantation CT scan) plotted over a 3D rendering of a presurgical MRI scan (Hermes et al. 2010; Branco et al. 2018b). Sensorimotor cortex electrodes with noisy or flat signal were removed from further analysis. For the remaining electrodes, line noise (50 Hz) and harmonics thereof were removed and common average re-referencing was applied. Audio recordings of the subject’s pronunciation were made during the task, to identify the voice onsets and offsets and to be able to correct for possible differences in behavioral performance (see below). Voice onset and offset were determined for each vowel pronunciation, as described previously (Salari et al. 2018). Shortly, these time points were first automatically determined using a vowel detection algorithm (Hermes 1990), which was adjusted by Hermes to also detect vowel offsets. Subsequently, we corrected the on- & offsets if necessary (due to background noise for instance) using Praat software (Boersma 2002).

Matlab software (The Mathworks, Inc., Natick, MA, USA) was used for data analysis, unless specified otherwise. For all sensorimotor electrodes, the high frequency band (75–135 Hz) power was computed per sample point using a Gabor wavelet (Bruns 2004) for all frequencies between 75 and 135 Hz in bins of 1 Hz with a full width half maximum (fwhm) of four wavelets per frequency. Subsequently, a log transformation (10 × log10) was applied and these results were then averaged (over frequencies) to create the HFB power signal. These signals were normalized and subsequently smoothed with a moving average window (centered around the sample point) of 0.1 s. This smoothing setting has been shown to be within the optimal range for accurate classification of phonemes (Branco et al. 2018a), and we used it to preserve the individual peaks per repetition in the data while reducing noise. The data from the two runs were concatenated.

Analysis of ECoG data was conducted in two steps. First, electrodes were identified and selected for further analysis based on their response to the task. Then signals from these electrodes were interrogated for vowel repetition effects.

### Electrode Selection

**Fig. 1:**
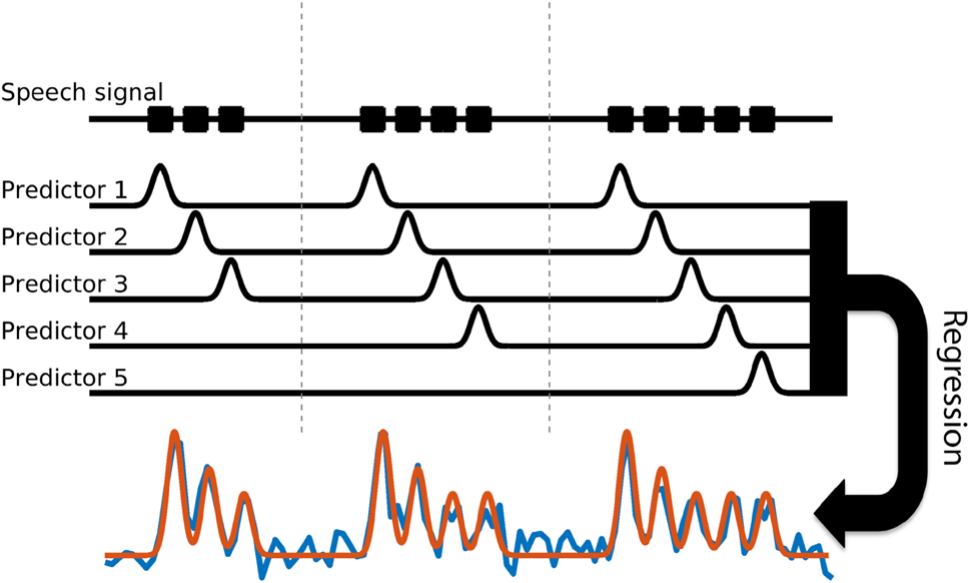
Model creation for electrode selection and deriving peak timings. A visual representation of the method to create from the signal (shown in blue) the model (shown in red) that was used to select significant electrodes and to determine the peak timings. Each predictor represents a transient response to one of the possible vowel production repetitions and are created by convolving a Gaussian function to an impulse at voice onset. The model was created by a regression of the five predictors to the signal. The model was used to find electrodes with a significant response to the task and to find the timing of (potential) peaks. The latter was done by shifting the predictors and repeating the regression until the best fit was found with the data

**Fig. 2:**
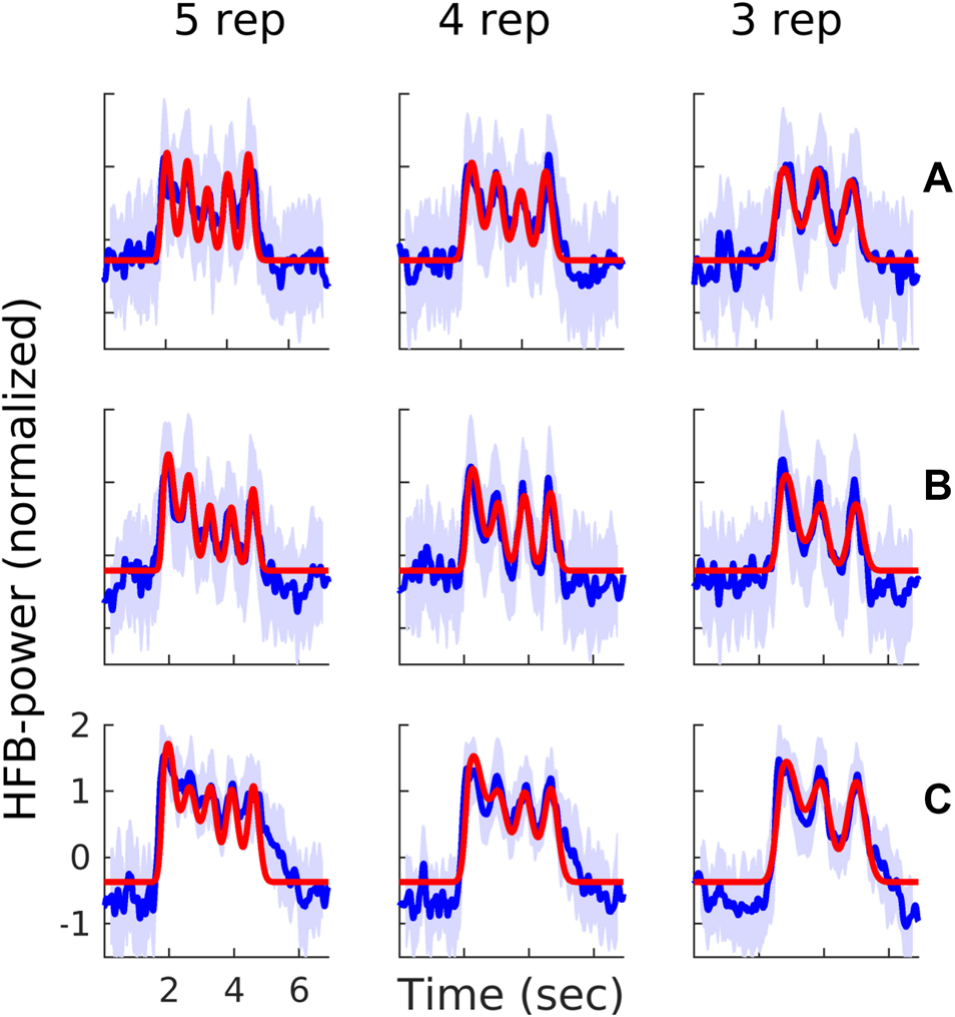
Data and model examples. Each row shows for one subject (**A**–**C**), an example of the response of one electrode in blue (average over trials, with shading indicating the standard deviation), with the three different production rates separated in columns (fastest on the left and slowest on the right). In red, the model that best fitted that specific electrode’s response is shown. On the x-axis, time is indicated in seconds after the first cue. On the y-axis, the normalized HFB-power is indicated

For each electrode, we determined whether it was responsive to the task. To that purpose, we modeled the neural signal by performing a regression analysis on the whole time series. Five predictors were used in this study, each representing a transient response to one of the possible repetition numbers (max 5). Predictor 1 represents the response to all first pronunciations, the second predictor to all second pronunciations etcetera. The fourth and fifth predictors had only (predicted) responses during the trials in which there actually was a fourth and/or fifth pronunciation (Fig. 1). The predictors were created by convolving a Gaussian function with an impulse function that indicated when a vowel was spoken. The full width at half maximum of the Gaussians were determined for each subject separately, as follows. First, for each electrode that had, in the trials of the slowest repetition rate, a maximum peak response higher than 1 standard deviation above the mean of the signal, we estimated the fwhm of that peak. The mean fwhm over electrodes was then used as the fwhm for the Gaussian peak of the model for the slowest production rate. For the two faster production rates, this value was adjusted to match those repetition rates by dividing it by the repetition frequency. We used the slowest repetition rate for the fwhm estimation under the assumption that this is least ‘contaminated’ with activity of other utterances. The fwhm values of the three subjects were, respectively, 0.59, 0.51 and 0.65 s for the 1 Hz repetition rate (leading to a 0.35, 0.30 and 0.39 s fwhm for the 1.66 Hz repetition rate and 0.44, 0.38 and 0.49 s fwhm for the 1.33 Hz repetition rate). Visual inspection showed that using these Gaussian widths, the neural activity could be accurately modeled for all subjects (Fig. 2). Subsequently, since it is known that the HFB response onset of different areas in the brain can occur at different time-points relative to the overt motor action (Crone et al. 1998; Coudé et al. 2011; Hermes et al. 2012b; Bouchard et al. 2013), we shifted the timing of the model peaks and repeated the regression until an optimal fit was found with the data. Timing shifts ranged from 0.5 s before voice onset time to 0.5 s after voice onset, in 0.1 s increments. An electrode was considered significantly responsive to the task if it was significantly explained by the best fitting model and if the average response over trials was an *increase* in power associated with at least the first vowel production (for all three repetition rates). For subject A–C, a total number of 14, 28 and 59 electrodes were significantly active, respectively. All other electrodes were considered not-responsive (NR). Statistical analysis was conducted using analysis of variance (ANOVA; α = 0.05, false discovery rate corrected), similar as in Salari et al. (2018). For this analysis, normally, each sample point is assumed to be independent and is used as a degree of freedom. The degrees of freedom (DF) value relates to the number of independent observations but since consecutive sample points are not independent (due to the Gabor wavelet power conversion) we counted every 0.5 s of data as an independent sample point to not overestimate the degrees of freedom. Note that, even though the data do not necessarily meet all the assumptions for parametric testing (as discussed above), inspection of the data showed that the current analysis was useful for selecting task related electrodes.

### Repetition Effects

Only the significantly responsive electrodes were used for further analysis. For these electrodes, we determined the HFB power peak amplitude for every vowel production. Since the HFB response peak timing, with respect to voice onset, could be different for each electrode we used the shift of the model that explained the data best as the determinant for timing of the HFB response peak with respect to voice onset. Each peak amplitude was determined by taking the median of the HFB signal in a window of 0.1 s before and 0.1 s after the determined peak timing. We used this value instead of the maximum value, to prevent possible noise peaks in the data to affect the results.

### Correction for Performance Differences

To investigate if the duration of vowel production was influenced by the repetition rate, we performed an ANOVA for each subject with pronunciation duration (derived from the audio signal) as dependent variable and production rate as independent variable. Furthermore, we corrected for possible differences in HFB response peak amplitudes that might be caused by differences in pronunciation between repetitions. We derived four behavioral performance measures, namely (1) sound intensity, (2) lip aperture, (3) lip movement and (4) lip velocity, for this correction. Sound intensity was calculated by taking the envelope of the normalized audio signal that was recorded during the task, using the absolute value of the Hilbert transform. The sound intensity was normalized per run to make measures from different sessions comparable. Normalization (of each run) was based on the mean and standard deviation of a silent part of that run. This envelope was then smoothed with a moving window of 0.05 s and down sampled to 600 Hz. Lip aperture was measured by analyzing video footage of the subjects while they performed the task. For each repetition, the mean distance (in pixels) between the lips was calculated for the video frames corresponding the pronunciation. Lip movements were calculated in a similar way but the video frames during silent parts just before each pronunciation were now used. The difference between the lip aperture during silence and the upcoming pronunciation served as a measure of lip movement. Lip velocity was calculated by taking the derivative of the lip positions during the silent part before each vowel production and subsequently taking the maximum value thereof. The lip position for each analyzed frame was normalized, per run. This was done by subtracting from each lip position sample, the mean number of pixels between the lips (over analyzed frames) and dividing this by the standard deviation (of pixels over time). To see if any of the measures could explain possible differences in the brain signals, we calculated the correlation value of each of these measures with the HFB response peak amplitudes for all included electrodes. Furthermore, a principal component analysis (PCA) was performed on these measures to dissect covariance among the different measures. The principal components were used as predictors of the HFB response peak amplitudes in a regression analysis, per electrode, the result of which was subtracted from the actual HFB response peak amplitudes to regress out any performance effects on the brain data. Outliers in the HFB response peak amplitudes were disregarded and outliers in the PCA values were replaced by the average value of that component. Outliers were determined by using the ‘isoutlier’ function from matlab. See Supplementary Figure S1 for an indication of the variance in brain data and behavioral measures and their relation before and after correction.

Since we could not measure the tongue position in the patient subjects, we did not correct for possible differences therein. However, after the current study we repeated the task with five healthy volunteers (who signed informed consent, median age: 26 years, range 22–31 years, 1 female) and recorded their tongue position using ultrasound measures. A total of 114 echo pulse scan lines were recorded at 60.11 frames per second at a depth of 90 mm with a EchoBlaster 128 ultrasound machine. The probe was stabilized using an ultrasound headset (Articulate Instruments Ltd 2008). The data were analyzed with Articulate Assistant Advanced software (Articulate Instruments Ltd 2012). We then evaluated whether repeated vowel production caused systematic changes in tongue movements.

After correction for performance, the peak amplitudes of all included electrodes were averaged and grouped by repetition number (1–3, 4 or 5) for each repetition rate separately. Subsequently, for each rate an ANOVA was performed with repetition number as independent variable and HFB response amplitude as dependent variable, to see if there was a significant difference in HFB-amplitude between repetition numbers. The result of this step was used as an indication of whether there was an influence of previous productions of the same vowel on subsequent productions. Since the slowest production rate only contained three repetitions, the ANOVA was performed on the first two and the last repetition only, for all production rates. Hermes et al. (2012b) suggested that a non-linear function in the form of a × (1/x) + bx + c best fitted their results with respect to the shape of the HFB response during finger movements. For visualization of the response profile, we fitted this function with the current data. Furthermore, since those authors found that for finger movements the HFB profile was dependent on movement rate, we compared the HFB response profiles of the three different repetition rates using an ANOVA. The repetition rate was used as independent variable and the HFB response peak amplitude was used as dependent variable (each repetition rate group consisted of the amplitudes of the first, second and last repetitions combined). Note that also in this step only the first, second and last repetitions were used to allow for comparison across rates.

### HFB Response Profiles

Based on previous research (Hermes et al. 2012b; Salari et al. 2018) and on inspection of the data, five models were defined to describe the HFB response profiles of the included electrodes. Electrodes could show (1) high activity for the first vowel production followed by a ‘non-linear decrease’ (NLD) of activity for the remaining productions, (2) high activity for the ‘first production’ (FP) but none or very little activity for the remaining productions, (3) high activity for the first and last production with a lower response for the productions in between, in the form of a ‘u-shape’ (US), (4) linearly decreasing (LD) activity over productions, or (5) activity could be equally responsive (ER) to all productions. Each electrode was classified as one of these response profiles for each repetition rate separately, by regressing three predictors to the HFB amplitude data of each electrode. Only three predictors were necessary to describe these five profiles as will be explained below. The first predictor models a NLD and a FP profile in a simplified way, with the first peak higher than the other peaks, and the other peaks being more or less equally high. For both the NLD and the FP profile the predictor was [1 0 0 0 0], [1 0 0 0] or [1 0 0] for a five, four and three repetitions trial respectively. If the intercept of the regression was significantly above zero (α = 0.05), the whole predictor would be moved up. In that situation, there would be a response present for all repetitions, which differentiates the NLD from the FP profile. The second predictor characterized the US model, (i.e., [1 0 0 0 1], [1 0 0 1] or [1 0 1]). The third predictor represented the LD model, (i.e., [1 0.75 0.5 0.25 0], [1 0.67 0.33 0] or [1 0.5 0]). Note that the slope of this linear predictor was not fixed as the beta and intercept value of the regression determined the slope. The predictor with the highest correlation to the data was chosen as the best fit. Subsequently, we tested if this predictor could significantly explain the amplitude response, based on the beta value from the regression analysis (α = 0.05). If an electrode was significant for the best fitting profile (i.e. NDL, FP, US or LD) it was classified as such. If none of the models were significant (and the electrode therefore did not show any difference between the response amplitudes of the repetitions), an electrode was assigned to the ER profile.

We determined, per repetition rate, the percentage of electrodes that belonged to each profile, and evaluated effects of production rate on the number of electrodes per profile.

To investigate the presence of an anatomical organization of particular response profiles within the sensorimotor cortex (i.e., whether some profiles are more prominent in specific sensorimotor regions than others), we determined for each electrode if it was classified as the same profile more than once (out of three repetition rates). If so, this profile was considered the most prominent profile for that electrode. We visualized the distribution of these most prominent response profiles on a 3D rendering of the subject’s brain as described in (Hermes et al. 2010; Branco et al. 2018b).

## Results

### Task Performance and Behavioral/Acoustic Measures

The task was performed well by all subjects, although subject C showed some difficulties during the first run. For subjects A & B, 7.7% (6/78) of the trials were disregarded due to an incorrect response and for subject C this was 35.9% (28/78). For the trials performed accurately (i.e. with the correct number of repetitions), the intended and performed repetition rates did not differ much (see Table 1). Subject A produced the vowels significantly slower than instructed for the fastest production rate, t(83) = − 7.54, p < 0.001 and subject C produced them faster for the two fastest repetition rates, t(43) = 2.63, p = 0.01 and t(50) = 4.21, p < 0.001 respectively.

**Table 1:** Behavioral performance

Subject	A	B	C
Production rate deviation (s)			
5 reps	− 0.06* (0.07)	0.01 (0.14)	0.04* (0.11)
4 reps	− 0.01 (0.07)	0.03 (0.14)	0.09* (0.16)
3 reps	0.01 (0.12)	0.04 (0.20)	0.06 (0.18)
Speech durations (s)			
5 reps	0.19 (0.03)	0.27 (0.05)	0.23 (0.07)
4 reps	0.20 (0.03)	0.27 (0.04)	0.21 (0.06)
3 reps	0.20 (0.03)	0.27 (0.04)	0.27 (0.07)
ANOVA	F(2,281) = 5.15, p = 0.006	F(2,281) = 1.17, p = 0.31	F(2,186) = 14.13, p < 0.001

In the top section, the mean and standard deviation of production rate deviations (instructed minus performed rate) over pronunciations are shown in seconds. The asterisk indicates a significant difference between intended and performed production rate. A negative value indicates that vowels were repeated slower than intended. In the lower section, the mean and standard deviation of speech duration is shown per production rate, for all subjects. Discarded trials were not included here

For subject A & C there was a significant difference between vowel production durations for the three different repetition rates after Bonferroni correction (α = 0.05), F(2,281) = 5.15, p = 0.006 and F(2,186) = 14.13, p < 0.001 respectively, see Table 1. For subject B, the vowel production durations did not differ significantly. Since the difference for subject A is relatively small (only 0.01 s), and there is no significant difference for subject B, these results suggest that there was not a strong overall difference between vowel production duration for the three repetition rates for these two subjects.

The derived behavioral performance measures (sound intensity, lip aperture, lip movement and lip velocity) did not correlate with the brain signal peak amplitudes for most electrodes (see Table 2 for the mean correlation over electrodes) in subjects A & B. In fact, for subject A, none of the electrodes showed a significant correlation to any of the measures. For subject B, only 17.86% (5/28) of the included electrodes showed a significant (α = 0.05, FDR corrected) correlation of HFB signal amplitude with sound intensity (mean r = 0.36, SD = 0.09). For subject C, many electrodes did show a significant correlation with the lip measures; 11.86% (7/59, mean r = 0.26, SD = 0.05) with lip position, 74.58% (44/59, mean r = 0.36, SD = 0.09) with lip movement, and 47.46% (28/59, mean r = 0.30, SD = 0.06) with lip velocity. Note that we correct for these effects in our ECoG analyses, see Supplementary Figure S1. This figure shows that most of the signal variability due to for instance sound intensity (see subject B) or lip movement (see subject C) is reduced by the correction we applied and will not have contributed to the results presented in the paper.

**Table 2:** Correlation between the performance measures and HFB peak amplitudes

Subject	A	B	C
Correlation			
Sound intensity	0.04 (0.08)	0.15 (0.14)	0.02 (0.08)
Lip aperture	0.01 (0.07)	− 0.09 (0.08)	0.13 (0.08)
Lip movement	0.05 (0.07)	0.02 (0.07)	0.31 (0.12)
Lip velocity	0.06 (0.06)	0.01 (0.05)	0.21 (0.10)

Values are the mean and standard deviation over included electrodes (therefore no p-values are shown)

### Electrode Selection and Peak Timing Models

The models used to select significant electrodes and to determine the peak timings showed an accurate correspondence to the HFB response signals (see Fig. 2), with an average of 62% (SD = 12), 67% (SD = 11) and 73% (SD = 12) variance explained for the included electrodes, of subjects A–C respectively.

### Average HFB Peak Profile During Vowel Repetitions

**Fig. 3:**
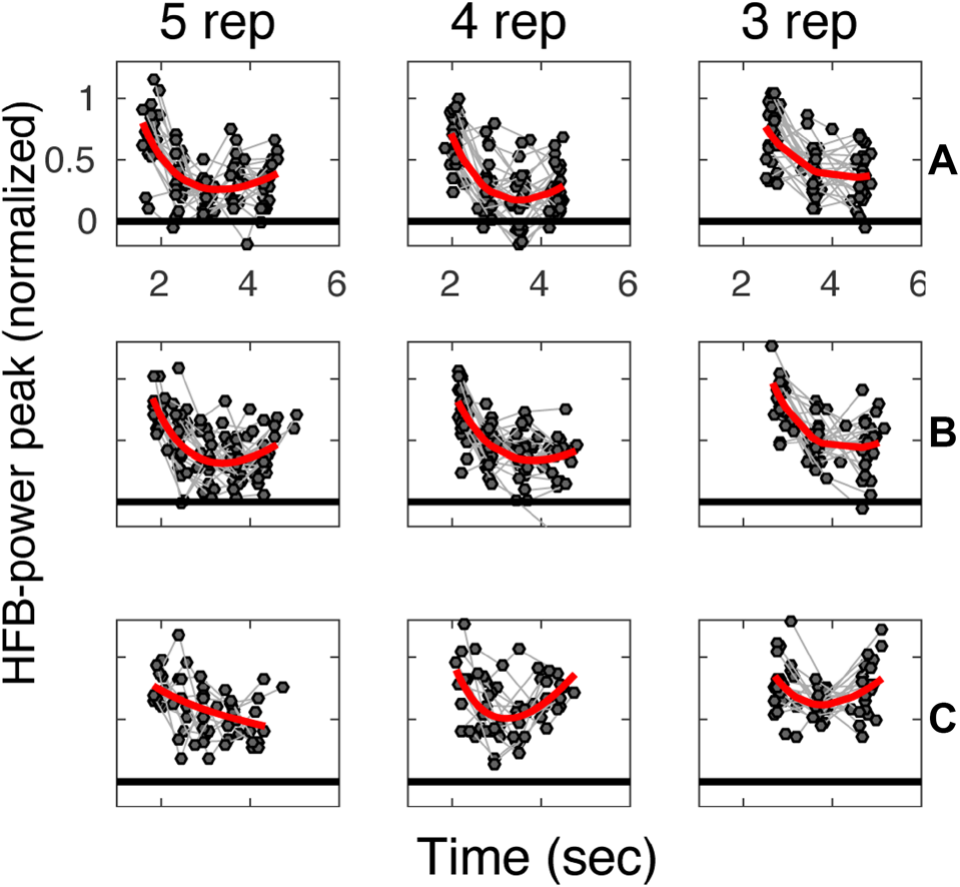
The average HFB profile for different vowel repetition rates. Each panel shows for one subject (**A**–**C**), the average profile of the HFB peak response for each vowel production averaged over included electrodes (marked by gray circles, pronunciations of the same trial are connected by gray lines). The three different production rates are separated in columns (fastest on the left and slowest on the right). A non-linear line, shown in red, was fitted to the data for visualization and for comparison with previous studies of hand movements (Hermes et al. 2012b). On the x-axis, time is indicated in seconds after the first cue and on the y-axis the normalized HFB-power is indicated

In general, the first vowel production of a trial was associated with a larger HFB peak amplitude than subsequent pronunciations (Fig. 3). For all subjects, the mean HFB response peak amplitudes over all significant sensorimotor electrodes differed significantly between repetitions for almost all repetition rates (Table 3, note that we used the first two and the last repetition for all rates). For subject C, there was no significant difference during the three repetitions condition.

**Table 3:** The ANOVA results per production rate and per subject, testing for differences between HFB peak amplitudes over repetitions

Subject	A	B	C
5 reps	F(2,59) = 20.61, p < 0.001	F(2,62) = 17.28, p < 0.001	F(2,30) = 4.69 p = 0.02
4 reps	F(2,73) = 25.63, p < 0.001	F(2,68) = 24.73, p < 0.001	F(2,45) = 6.26, p < 0.001
3 reps	F(2,71) = 19.49, p < 0.001	F(2,74) = 28.43, p < 0.001	F(2,60) = 3.03, p = 0.06

The dependent variable was the mean (over included electrodes) HFB peak amplitudes and the independent variable was the repetition number. Significance indicates that the HFB peak amplitudes are significantly different between repetitions

**Fig. 4:**
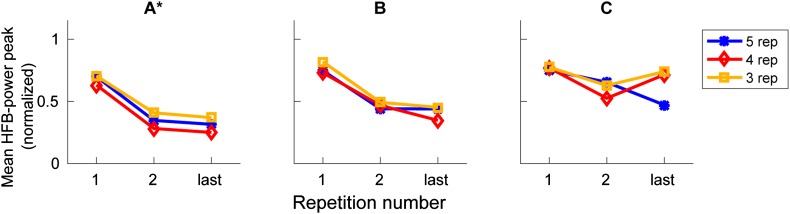
Comparison of HFB profiles between different production rates. Each panel shows for one subject (**A**–**C**), the HFB amplitude per repetition number, averaged over included electrodes and over trials. The three different production rates are indicated in blue, red and yellow (for the first two and the last repetition). On the x-axis, the repetition number is indicated and on the y-axis the normalized HFB-power is indicated. A significant difference between conditions is indicated by an asterisk

We investigated whether certain repetition rates were associated with a stronger average decrease in amplitude than other repetition rates. Only subject A showed a significant difference between peak amplitudes over production rates (Fig. 4), F(2,209) = 3.27, p = 0.04.

### Electrode HFB Peak Profiles

**Fig. 5:**
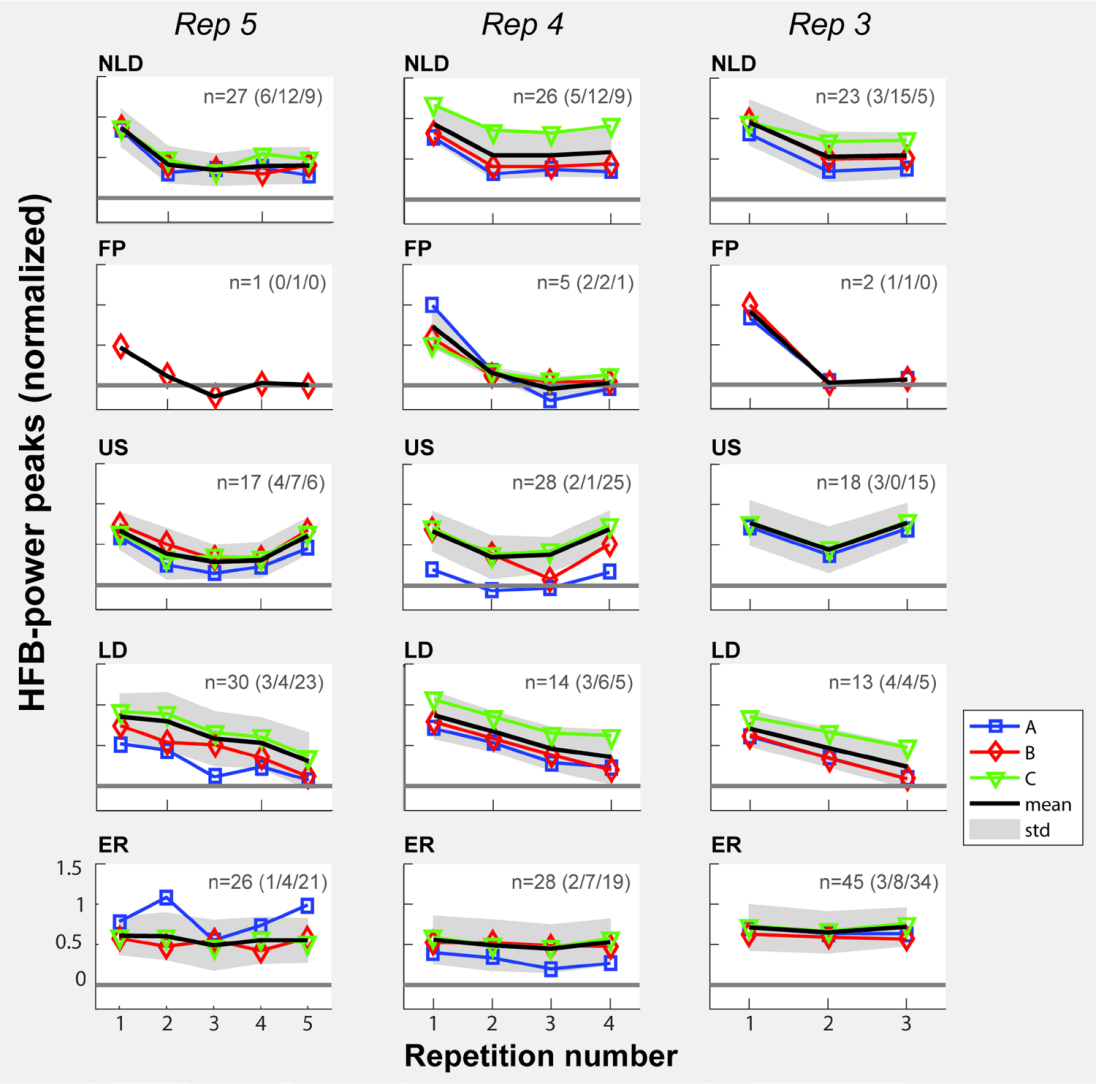
Representation of all response profiles. Responses were ‘non-linear decreasing’ (NLD) ‘first production responsive only’ (FP), ‘u-shaped’ (US), ‘linearly decreasing’ (LD) or ‘equally responsive’ (ER). Each row indicates the average response (per subject in color and for all subjects in black) of all electrodes that belonged to one profile. The number of electrodes on which the mean response was based is indicated by ‘n’ and the number of electrodes each subject contributed is indicated by the numbers in brackets for subject A–C respectively. Columns separate the different production rates. The standard deviation of the response over subjects is shown by shading. On the x-axis, the repetition number is indicated and on the y-axis the normalized HFB-power is indicated

**Fig. 6:**
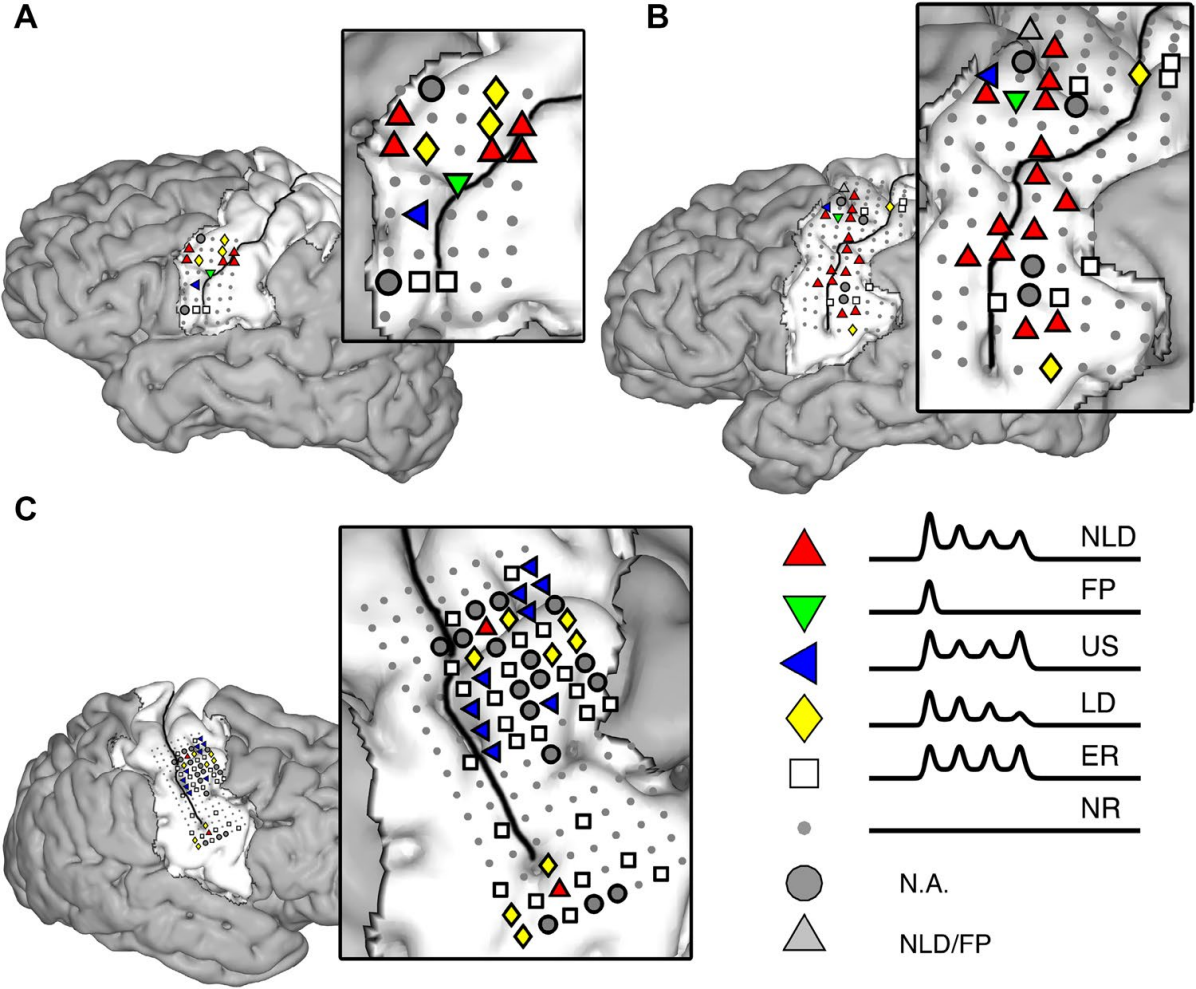
Electrode localization. For each subject, the locations of all analyzed electrodes are indicated. Small gray dots indicate non-responsive (NR) electrodes. The other symbols indicate, per electrode, the most prominent response profile, which was determined by classifying the HFB responses for each production rate into one of five response profiles (for abbreviation clarification of the response profiles see Fig. 5). If an electrode was classified as the same response profile for at least two out of the three production rates, this was considered the most prominent response profile. Gray circles (see N.A. in legend) indicate electrodes that did not have a prominent response (i.e. where the responses were different for each production rate). The difference between the NLD and FP response predictor was an intercept difference and therefore light gray triangles (see NLD/FP in legend) indicate electrodes which had different responses for each repetition rate but which were classified once as NLD and once as FP

Each included electrode was classified as belonging to one of five response profiles (Fig. 5) based on the development of the peak amplitude over repetitions, for each repetition rate separately. In general, the NLD was the most frequent response profile for subjects A & B. For subject C, the US and ER responses were most frequent. None of the response profiles showed a clear anatomical clustering (Fig. 6).

**Fig. 7:**
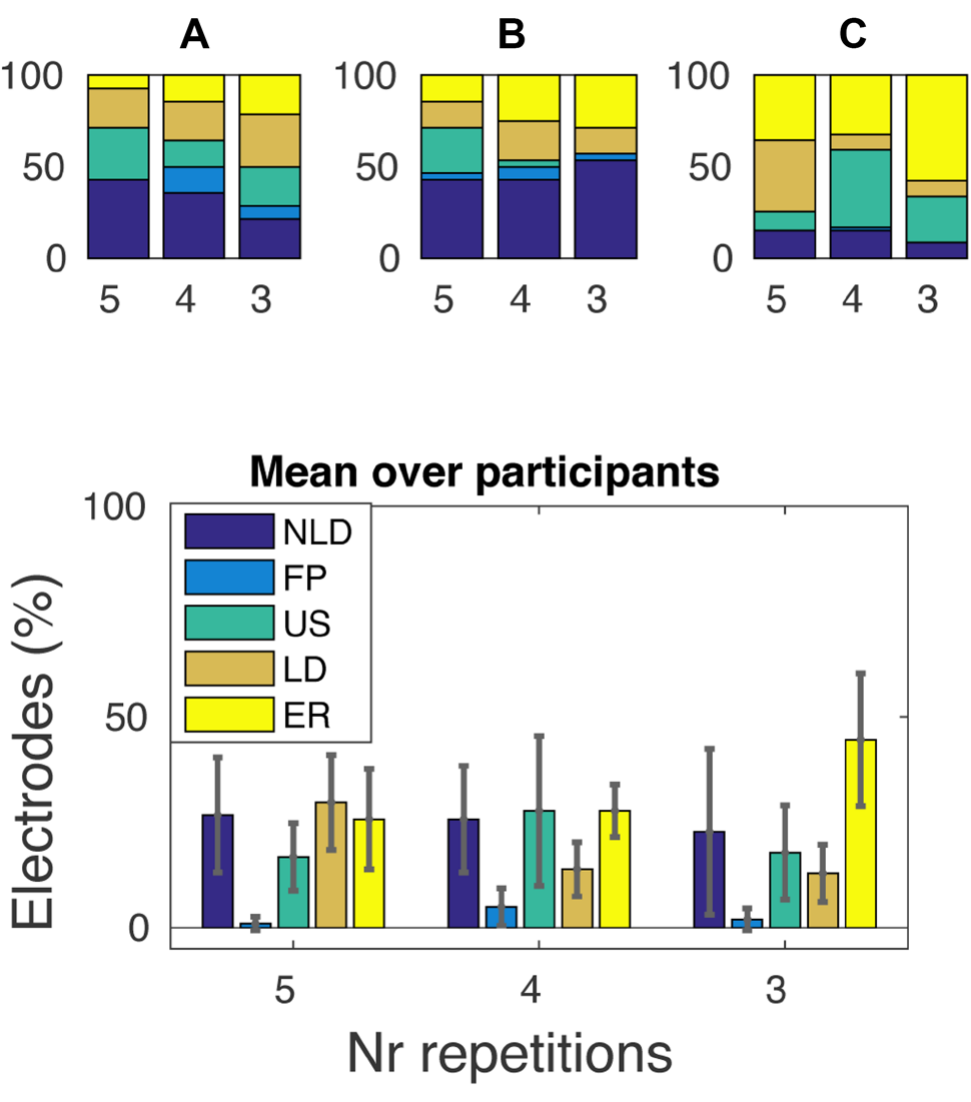
Number of electrodes for each profile. In the upper panels, the percentage of included electrodes belonging to a profile (indicated by color) is shown for all subjects and production rates (indicated on the x-axis). On the y-axis, the percentage of electrodes is indicated. For subject **A**–**C** a total number of 14, 28 and 59 electrodes were significantly active, respectively, which corresponds to the 100% value for each subject. The lower panel shows the weighted mean and standard deviation values over subjects

We investigated whether there was a general change in the number of electrodes per profile depending on repetition rate (Fig. 7). Although no statistical conclusions can be drawn from the results with the current number of subjects, there was an overall trend for an increasing number of ER electrodes with decreasing frequency rate. For subjects A, B and C, respectively, 15.38% (2/13), 20.83% (5/24) and 52.63% (20/38) of all electrodes that showed a repetition effect in the five repetitions condition convert to ER in the three repetitions condition.

### Tongue Movements in Healthy Volunteers

**Fig. 8:**
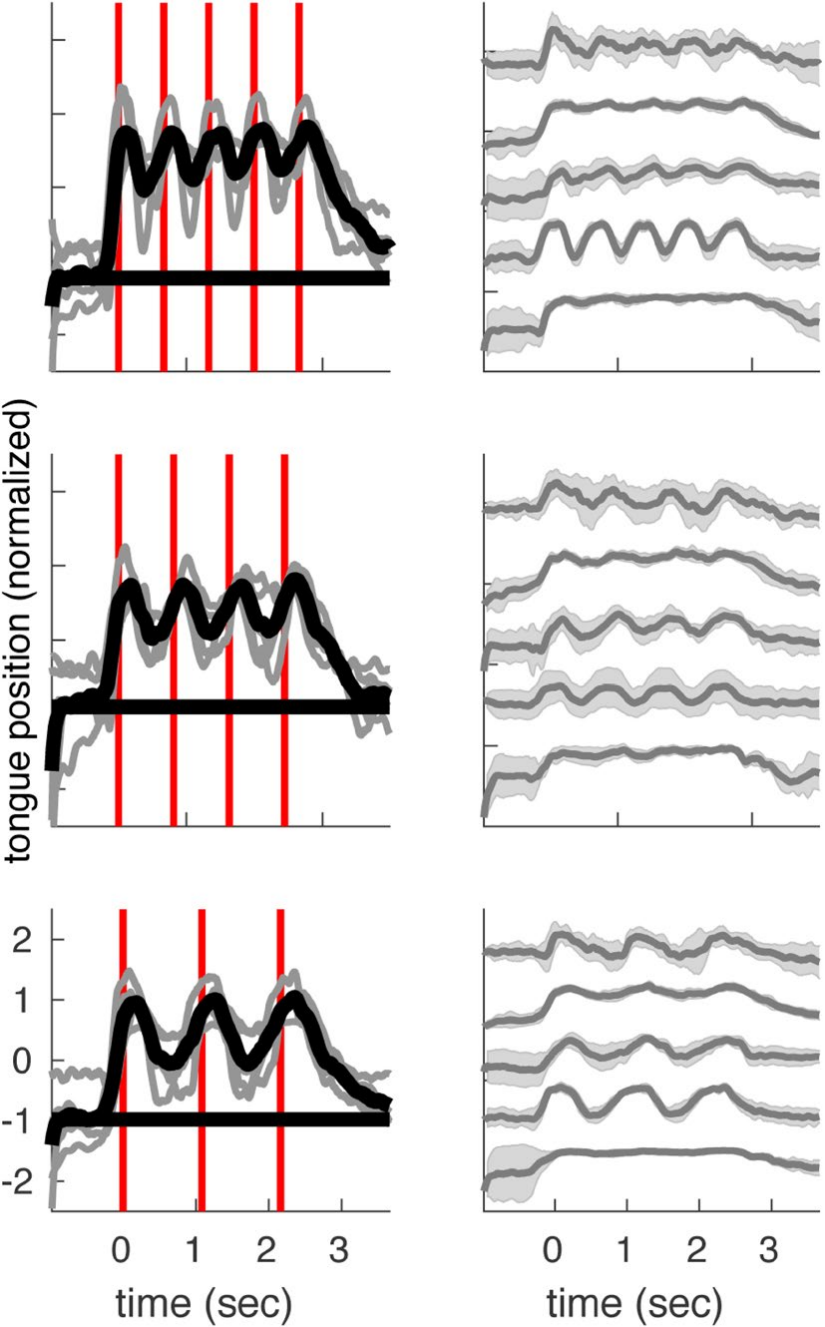
Tongue positions. The average tongue position height (normalized) of five healthy volunteers is shown on the y-axis. The part of the tongue that moved the most with the task is shown. On the x-axis, time is indicated in seconds. Red vertical lines indicate voice onsets. The three different repetition rates are shown from top to bottom. The data was corrected for differences in pronunciation timing and was subsequently averaged per subject. Gray lines indicate the average tongue position of the individual volunteers (shading indicates the standard deviation) and the black line in the left panel represents the average thereof

The results from the tongue movement measures indicated that 4 subjects did not show much difference in tongue position over the different repetitions and one subject showed a slightly higher tongue position for the first vowel production compared to subsequent repetitions for the two fastest repetition rates (Fig. 8). Whether or not people returned their tongue to the rest position in-between repetitions was quite different over subjects.

## Discussion

The effect of movement repetition on the sensorimotor HFB response during vowel production was investigated using a simplified speech task with controlled speed of repetition. The HFB signal from high-density electrode grids was evaluated in three epilepsy patients undergoing a surgical procedure for epilepsy diagnosis.

We show that sensorimotor activity related to discrete speech movements is influenced by previous speech movements when spaced a second (or less) apart. Averaged across electrodes, the HFB-response of sensorimotor cortex had a similar amplitude between different production rates but did not show equal amplitudes over the course of repetitions (Fig. 3). This was seen for all subjects and all tested production rates (1–1.66 Hz; except for one instance in subject C where the effect was near-significant).

The data suggest that the HFB-amplitude is not linearly related to motor output since amplitudes mainly decline non-linearly for repeated vowel productions. The analysis included a correction for the small variations in sound intensity, lip aperture, lip movement and lip velocity, making it unlikely that this finding can be explained by differences in performance over repetitions. However, movements of the tongue could not be measured (discussed below).

The results for speech movements are in agreement with earlier electrophysiological and fMRI data that report a repetition effect for finger movements (Hermes et al. 2012b; Siero et al. 2013). We extend these by showing that complicated movements such as those involved in speech show a decline in HFB response when repeated at a frequency of 1 Hz or higher. Furthermore, our data suggest a tipping-point between 1 and 2 s (the production rate at which the HFB amplitude decline disappears) since the repetition effect was still visible for repetitions 1 s apart but was no longer visible after 2 s, the time approximately between the last pronunciation of a trial and the first pronunciation of the following trial, as indicated by the recovery of a high amplitude for each first pronunciation of a trial.

Across electrodes, the non-linear decrease (NLD) profile was dominant for subject A & B. Other response profiles were observed, but less frequently in these subjects. For subject C, the US and ER responses were most frequent. For all subjects, the number of electrodes with an equally responsive (ER) profile tended to increase with decreasing vowel production rate. Considering the earlier discussion on the tipping-point, it could be speculated that different cortical patches in sensorimotor cortex exhibit different tipping points, which would cause more electrodes to display the repetition effect as vowel production rate increases. Note however, that the total number of pronunciations and therewith the number of data points, is different for each repetition rate, which could make the statistical chance to find a particular response profile unequal between repetition rates. Therefore, we cannot fully exclude the possibility that the tipping point effect may be caused partly by an unequal number of data points between repetition rates.

The current results did not indicate a clear anatomical organization with respect to the different response profiles although some clustering seemed to be present (most clearly visible in subject B). Please note that, even though we used HD electrode grids, individual electrodes are still 3 or 4 mm apart. Therefore, the spatial sampling is somewhat sparse compared to for instance high field fMRI recordings. Possibly, repeating the experiment with even higher spatial sampling may reveal spatial organization with respect to the repetition effect.

### Neural Underpinnings of the Repetition Effect

There are several phenomena that may account for the decrease in HFB-power observed with repeated speech movements. It may be speculated that some articulators which we did not correct for, moved more for the first pronunciation than for subsequent pronunciations. Analysis of tongue movements during the same task in healthy volunteers revealed quite constant tongue positions over repetitions within subjects but also revealed variations in tongue movements between subjects, ranging from full contraction and relaxation for each repetition to a fixed tongue position throughout repetitions (Fig. 8). Previous research has suggested that not only articulator position but also features related to articulator movements (such as velocity) are represented in the sensorimotor cortex (Conant et al. 2018). In case the tongue may not return to its rest position between repetitions, the first utterance may be associated with more activation than the subsequent productions as the articulator movement is then largest for the first pronunciation and smaller for the subsequent ones. This may also explain why the last repetition sometimes showed an increase in activity compared to its predecessor(s) as the tongue has to return to its rest position. Furthermore, it can be speculated, that between phonemes the musculature used for the production is not fully at rest (in anticipation of the next production), even if the articulator position between phonemes is close to the rest position. In this case, one could see the full sequence of repetitions as one, albeit complex, movement with an onset and an offset. Since various reports have shown a neural response at movement offset (Ball et al. 2008; Hermes et al. 2012b; Salari et al. 2018), neural activity at the end of a sequence may also be attributed to the movement towards full rest. However, this cannot explain all the found response profiles. Hence, our findings cannot be fully explained by differences in tongue movements between repetitions and are therefore also in line with the existence of a non-linearity between motor output and neural activity during repeated speech-movements that are spaced closely apart. Since HFB power is thought to be associated with neural firing (Manning et al. 2009; Miller et al. 2009; Ray and Maunsell 2011), a decrease in HFB power as observed here may suggest that fewer neurons are involved in subsequent motor acts, (see Hermes et al. 2012b for a similar interpretation), or that the same neurons fire less frequently. Indeed, repetition suppression effects have been found for other modalities than motor execution. Suppression during repeated visual stimulation has for example been attributed to a reduction of neural excitability for repeated stimuli (see Grill-Spector et al. 2006 for an interesting discussion on the possible mechanisms behind a reduction of neural activity for repeated stimuli and the possible function this may have).

Furthermore, repetition suppression effects for repeated speech have also been found in other areas than the sensorimotor cortex and may be involved in motor planning. Previous fMRI research in the left posterior inferior frontal gyrus, has shown for instance, a repetition suppression effect which is related to the degree of shared phonological features (such as voicing or manner of articulation) over the course of repeated words (Okada et al. 2018). This suggests that similar phonological features during speech reduce activity in motor planning areas. It would be interesting to see if such motor planning effects of similar phonological features is related to the repetition effect we observed in the sensorimotor cortex as it has been suggested that repetition effects in some areas may affect the activity in other areas (Grill-Spector et al. 2006).

Furthermore, even though in the current study we focused on sensorimotor cortex activity, other areas such as the supplementary motor area (SMA), cerebellum, basal ganglia and premotor cortex, which are connected to the motor cortex, have been suggested to play an important role in the timing of speech production (Kotz and Schwartze 2010). It would therefore be interesting to investigate the role of those areas during repeated speech production and to see if they have an influence on the repetition effect.

### Neural Underpinnings of the Different Response Types

Although further research is needed to better understand the different response types we found, we may speculate about the possible underlying mechanisms. There are multiple theories on the mechanisms behind repetition suppression that may explain the current results. For instance, increased influx of potassium ions over the course of repetitions may lead to hyperpolarization of the cell membrane, causing a reduction in neural firing. If this effect is asymptotic, this may lead to a non-linear decrease. Another theory suggests that only neurons that are most specific to the task continue firing over repetitions. It could be that in some areas the number of task-specific neurons is higher than in others, which may lead to the different response types. If most of the neurons are task-specific, it would be likely that the responses are equal over repetitions (ER). If the ratio between task-specific neurons and task-unspecific neurons is high, the number of firing neurons may decrease initially and stabilize at some point, leading to a non-linear decrease (NLD or FP). With a lower ratio, the decrease may be linear, as the number of neurons that can stop firing is larger. As discussed earlier, some areas may be related to movements in two directions (Fetz et al. 1980; Soso and Fetz 1980), which may explain the u-shape response type (US), as at the end of the trial the articulators are likely to return to rest position, see for instance the tongue position data in Fig. 8. This may result in increased neural firing at the end of the trial. Besides these theories on the neural underpinnings of repetition suppression, there may be an alternative explanation for a higher response for the first vowel production. Some parts of the sensorimotor cortex may be involved in the planning of a motor sequence (Tanji and Evarts 1976) and it may be speculated that this could lead to more neural activity for the first production (i.e. beginning of a rhythmic sequence) or to only neural activity at the beginning of the sequence.

### Implications for Neural Based Speech Decoding

Our results are highly relevant for the development of sensorimotor-speech BCIs: systems that aim to decode (attempted) speech from sensorimotor brain signals. Classification of speech sounds based on sensorimotor activity has been shown before (Kellis et al. 2010; Brumberg et al. 2011; Mugler et al. 2014; Herff et al. 2015; Ramsey et al. 2017), but accuracy levels and degrees of freedom do not meet the standards for home-use by patients. It has been suggested however, that these systems are likely to benefit from the use of high-density electrode grids (Kellis et al. 2016; Ramsey et al. 2017). Classification of sensorimotor signals may also benefit from taking linguistic structures, such as syntax or likely word combinations, into consideration by incorporating a language corpus to the predictions (Herff et al. 2015, 2017). We postulate that a third factor needs to be taken into account for optimal decoding accuracy: previous speech movements. Since sensorimotor-speech BCIs try to find specific patterns in the brain signals that can consistently be linked to a specific sound, and use this information to determine which sound the user made (or tried to make), any effect of previous (attempted) utterances on the brain signal of a current utterance is important information. Sensorimotor-speech BCIs may therefore be improved when information about previously spoken sounds is incorporated in the decoding pipeline. The current study provides a method for creating models of HFB profiles related to vowel repetitions. Models like these may be used for creating a library of models related to variations in brain activity patterns associated with speech production and may potentially improve speech classification. It will be crucial to extend the current findings to more real-life application scenarios of sensorimotor-speech BCIs, and to investigate whether the results can be generalized to natural speech circumstances such as repeating the same phoneme within a word or over the course of words. Furthermore, since these BCI systems are intended for paralyzed subjects, it is essential to investigate if also repeated covert/attempted speech is associated with similar phenomena.

### Limitations & Future Work

One of the limitations of the current study is the small number of subjects. Yet all subjects show similar results (decreased activity for repeated vowel production) across all investigated production rates (except for one instance in subject C where the effect was near-significant) and our findings do correspond to that of previous studies for finger movements. Also, a larger range of production rates could have been more informative, notably to determine the tipping point for HFB response recovery. Third, we did not record articulator positions directly (except for the lips) and could therefore not correct for all variations in motor output. Fourth, in the current study we did not control for a possible effect of auditory stimulation on the cortical responses (by the subjects hearing their own voice during the task). However, since previous studies of other repeated movements (not involving speech or auditory stimulation) have shown similar results as the current study (e.g. Hermes et al. 2012b), we argue that it is likely that the repetition effect is more a sensorimotor cortex effect related to movements than to auditory stimulation. Finally, from our study it is not possible to determine whether the repetition effect is specific for the same phoneme, or could generalize to different phonemes following one another. This issue clearly warrants further investigation, as it is relevant for decoding speech where different phonemes are produced in sequence.

## Conclusion

We show that neural activity related to discrete repeated speech movements is influenced by previous speech movements spaced a second or less apart. The most prominent response profile for repeated speech movements is a non-linear decrease of neural activity over repetitions. These findings are of importance for the development of communication-brain-computer interfaces that use decoding of (overt or covert) speech.

## Electronic supplementary material

Below is the link to the electronic supplementary material.

Supplementary Figure S1—Correlation, for subject A-C, between four behavioral measures (sound intensity, lip position, lip movement and lip velocity) with the normalized brain signal peak amplitudes, averaged over electrodes, before (blue) and after correction (red) for behavioral measures. On the x-axis, the HFB signal peak amplitude is indicated and on the y-axis the behavioral measure. The correlation value (r) and significance value (p) are indicated above each plot in the corresponding color (TIF 7380 KB)
