# Supplementary figures and images for: Repeated Vowel Production Affects Features of Neural Activity in Sensorimotor Cortex
Source: Brain Topogr. 2018 Sep 20;32(1):97–110. doi: 10.1007/s10548-018-0673-4 (PMC6326960; doi:10.1007/s10548-018-0673-4)

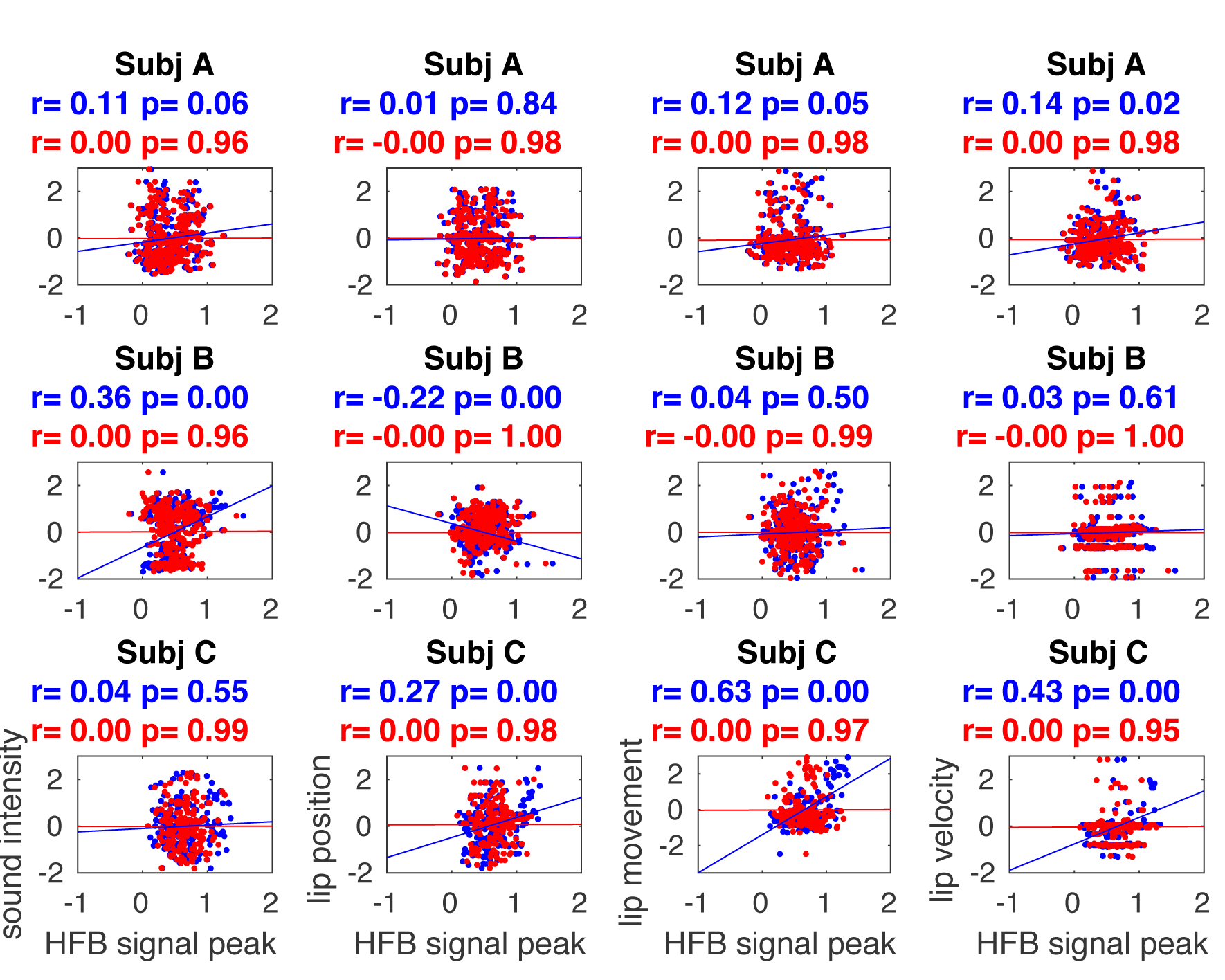

Supplement: Supplementary file 1 — Supplementary Figure S1—Correlation, for subject A-C, between four behavioral measures (sound intensity, lip position, lip movement and lip velocity) with the normalized brain signal peak amplitudes, averaged over electrodes, before (blue) and after correction (red) for behavioral measures. On the x-axis, the HFB signal peak amplitude is indicated and on the y-axis the behavioral measure. The correlation value (r) and significance value (p) are indicated above each plot in the corresponding color (TIF 7380 KB) [file 10548_2018_673_MOESM1_ESM.tif]
